# Environmental adversity and uncertainty favour cooperation

**DOI:** 10.1186/1471-2148-7-240

**Published:** 2007-11-30

**Authors:** Peter Andras, John Lazarus, Gilbert Roberts

**Affiliations:** 1 School of Computing Science, Newcastle University, Newcastle upon Tyne, UK; 2 Institute of Neuroscience, Newcastle University, Newcastle upon Tyne, UK

## Abstract

**Background:**

A major cornerstone of evolutionary biology theory is the explanation of the emergence of cooperation in communities of selfish individuals. There is an unexplained tendency in the plant and animal world – with examples from alpine plants, worms, fish, mole-rats, monkeys and humans – for cooperation to flourish where the environment is more adverse (harsher) or more unpredictable.

**Results:**

Using mathematical arguments and computer simulations we show that in more adverse environments individuals perceive their resources to be more unpredictable, and that this unpredictability favours cooperation. First we show analytically that in a more adverse environment the individual experiences greater perceived uncertainty. Second we show through a simulation study that more perceived uncertainty implies higher level of cooperation in communities of selfish individuals.

**Conclusion:**

This study captures the essential features of the natural examples: the positive impact of resource adversity or uncertainty on cooperation. These newly discovered connections between environmental adversity, uncertainty and cooperation help to explain the emergence and evolution of cooperation in animal and human societies.

## Background

The drive to understand the emergence of cooperation – actions of benefit to both actor and recipient – in communities of selfish individuals has generated a large body of theoretical and empirical research in recent decades [1-14]. This research focuses on the dynamics of interactions between individuals and pays relatively little attention to the effects of the environment. However, evidence is growing, in many taxa, that as the adversity (harshness) and uncertainty of the environment increase cooperation is enhanced and we present a model here that attempts to explain this phenomenon as an adaptive facultative response favoured by selection.

An organism's environment is more adverse if some quality such as resources, physical structure, climate, competitors, parasites or predators changes in such a way as to decrease darwinian fitness. Environmental adversity is species-specific, e.g. high temperature may be adverse for some organisms, but not for thermophilic bacteria. As an example of the uncertainty or unpredictability of the environment [15], feeding in a patchy area, where some places are rich in food and others barren, results in greater uncertainty of nutritional status compared to foraging where food is distributed homogeneously. Uncertainty can be measured as the variance of a distribution of environmental quality, and adversity as the mean [16]. Both adversity and uncertainty have been conceptualised as aspects of environmental 'risk' [17].

At many levels of life, from plants to human societies, cooperation thrives in conditions where the environment is most adverse. Plants at lower temperatures and higher altitudes, where abiotic stress is high, compete less and cooperate more with their neighbours [18]; nematodes *Caenorhabditis elegans *aggregate in response to stressors [19]; animals form more cohesive or larger groups, with consequent greater mutualistic benefits under greater predation risk [20-23]; mole-rats, a highly social species, delay dispersion more in arid than in mesic habitats [24]; human in-group solidarity is greatest when the group is under threat or in a harsh environment [25-28].

In humans there is also evidence for enhanced cooperation where the environment is more uncertain. This holds for common pool resource groups, such as fisheries [29], for communal sharing of hunted meat in various societies [30], and for sharing in laboratory experiments [31]. Examples of enhanced cooperation under adversity and uncertainty are discussed further by Andras & Lazarus [32].

We present a model to explain this increase in cooperation under conditions of adversity and uncertainty. The model has two parts. First, we show that adversity increases the organism's uncertainty in its resource level, uncertainty being measured as subjectively perceived resource variance (sub-section 2.1). We then show that resource uncertainty increases cooperation, using a multi-agent simulation (sub-section 2.2).

## Results and Discussion

### Adversity and uncertainty: analytical results

**Figure 1:**
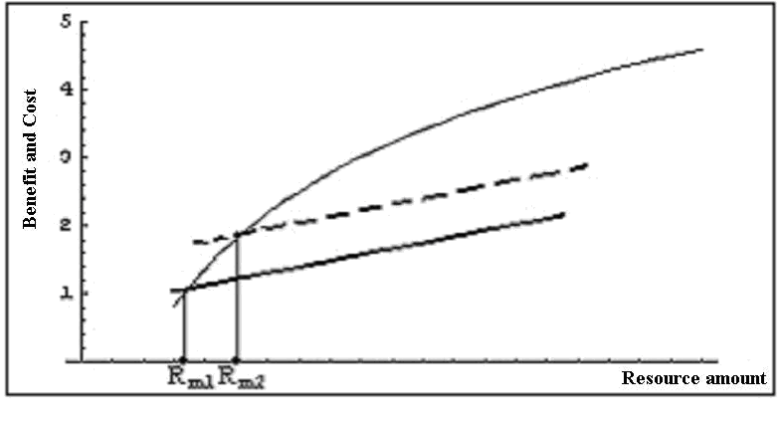
**The minimal acceptable level of resources**. The incremental fitness cost (assumed to be straight lines for simplicity) and benefit (curved line) are shown as a function of the resource amount gained, in a less adverse (continuous straight line) and a more adverse (segmented straight line) environment (the slope of the two lines is the same). Cost increases with resource amount since, optimally, more investment (cost) is made when the return (benefit) is greater. The resource amounts *R*_*m*1 _and *R*_*m*2 _are assumed to be the minimal acceptable levels of resources in the two environments.

We consider the influence of adversity on the minimal amount of a given resource that the individual finds acceptable for survival. We assume that the marginal fitness benefit of resource amounts decreases with the amount of resources the organism already has. For example, eating an extra food unit adds less to the fitness of a well fed animal than to the fitness of a hungry animal [32]. We also assume that the cost of acquiring resources increases monotonically with the amount of resources acquired. For example capturing a large prey takes more energy than capturing a small prey (see also Fig 1 legend for general argument). For the sake of simplicity we assume that the cost function is linear (although we note that in practice this may be depart from linearity in the sense that acquiring a certain biomass of resources in the form of five small prey items may be more expensive than acquiring the same biomass in the form of a single large prey item).

The cost of foraging for resources is assumed to increase with adversity. For example, energy lost to foraging will be more costly in colder environments, and time spent foraging (and not available for anti-predator vigilance) will be more costly in environments with greater predation risk. We are interested in finding the minimal level of a particular resource that it is profitable to exploit, considering resource costs imposed by the environment (i.e. the minimal amount for which the benefits of acquiring the resource are larger than the costs of acquiring the resource). We term this level of resource the minimal acceptable level of resource. Note that we are not looking for the optimal level of resources, i.e., where the marginal benefit equals the marginal cost.

We denote by *D*(*R*) the probability density function of the distribution of resources (*R *– the amount of resources, *R ≥ 0*) in an individual's environment (i.e. P(R1<R<R2)=∫R1R2D(R)dR
MathType@MTEF@5@5@+=feaafiart1ev1aaatCvAUfKttLearuWrP9MDH5MBPbIqV92AaeXatLxBI9gBaebbnrfifHhDYfgasaacPC6xNi=xH8viVGI8Gi=hEeeu0xXdbba9frFj0xb9qqpG0dXdb9aspeI8k8fiI+fsY=rqGqVepae9pg0db9vqaiVgFr0xfr=xfr=xc9adbaqaaeGacaGaaiaabeqaaeqabiWaaaGcbaGaemiuaaLaeiikaGIaemOuai1aaSbaaSqaaiabigdaXaqabaGccqGH8aapcqWGsbGucqGH8aapcqWGsbGudaWgaaWcbaGaeGOmaidabeaakiabcMcaPiabg2da9maapehabaGaemiraqKaeiikaGIaemOuaiLaeiykaKIaemizaqMaemOuaifaleaacqWGsbGudaWgaaadbaGaeGymaedabeaaaSqaaiabdkfasnaaBaaameaacqaIYaGmaeqaaaqdcqGHRiI8aaaa@4509@ is the probability that the amount of resources in a resource item or in a resource location is between the values *R*_1 _and *R*_2_). Let us assume that *R*_*m*1 _and *R*_*m*2 _are the minimum acceptable level of resource in a less adverse (1) and a more adverse (2) environment with the same resource distribution; we then get *R*_*m*1 _<*R*_*m*2 _(Figure 1). In other words, the minimum acceptable level of resources is greater in the more adverse environment. This prediction is supported by field studies of foraging in rodents, in which individuals select more profitable foods under environmental conditions – e.g. open habitat and moonlit nights – in which there is a greater risk from nocturnal predators [33-36]. (Animals under immediate predation risk, where anti-predator behaviour competes directly with foraging, trade-off the two concurrent demands and *reduce *selectivity in feeding in order to attend to the threat of predation [37-39], but this phenomenon is not relevant to the long-term response to adversity – and when prey are not under immediate threat of predation – with which we are concerned here. This distinction can explain the contrasting results of the two sets of studies.)

The minimum acceptable cut-off point means that all resources below that limit are considered by the organism to be equivalent to zero, accessing them being unprofitable. This allows us to derive a relationship between adversity and subjective resource uncertainty (i.e., the variance of the resource distribution after ignoring the resources below the minimum acceptable cut-off point – for example the variance of the distribution of sizes of acceptable food items, which are sufficiently big to be above the minimum acceptable cut-off point). Writing the formulae for the subjective variances of resources in the two environments we get:

V1=∫Rm1+∞R2D(R)dR−R¯12
MathType@MTEF@5@5@+=feaafiart1ev1aaatCvAUfKttLearuWrP9MDH5MBPbIqV92AaeXatLxBI9gBaebbnrfifHhDYfgasaacPC6xNi=xI8qiVKYPFjYdHaVhbbf9v8qqaqFr0xc9vqFj0dXdbba91qpepeI8k8fiI+fsY=rqGqVepae9pg0db9vqaiVgFr0xfr=xfr=xc9adbaqaaeGacaGaaiaabeqaaeqabiWaaaGcbaGaemOvay1aaSbaaSqaaiabigdaXaqabaGccqGH9aqpdaWdXbqaaiabdkfasnaaCaaaleqabaGaeGOmaidaaOGaemiraqKaeiikaGIaemOuaiLaeiykaKIaemizaqMaemOuaifaleaacqWGsbGudaWgaaadbaGaemyBa0MaeGymaedabeaaaSqaaiabgUcaRiabg6HiLcqdcqGHRiI8aOGaeyOeI0IafmOuaiLbaebadaqhaaWcbaGaeGymaedabaGaeGOmaidaaaaa@4505@

V2=∫Rm2+∞R2D(R)dR−R¯22
MathType@MTEF@5@5@+=feaafiart1ev1aaatCvAUfKttLearuWrP9MDH5MBPbIqV92AaeXatLxBI9gBaebbnrfifHhDYfgasaacPC6xNi=xI8qiVKYPFjYdHaVhbbf9v8qqaqFr0xc9vqFj0dXdbba91qpepeI8k8fiI+fsY=rqGqVepae9pg0db9vqaiVgFr0xfr=xfr=xc9adbaqaaeGacaGaaiaabeqaaeqabiWaaaGcbaGaemOvay1aaSbaaSqaaiabikdaYaqabaGccqGH9aqpdaWdXbqaaiabdkfasnaaCaaaleqabaGaeGOmaidaaOGaemiraqKaeiikaGIaemOuaiLaeiykaKIaemizaqMaemOuaifaleaacqWGsbGudaWgaaadbaGaemyBa0MaeGOmaidabeaaaSqaaiabgUcaRiabg6HiLcqdcqGHRiI8aOGaeyOeI0IafmOuaiLbaebadaqhaaWcbaGaeGOmaidabaGaeGOmaidaaaaa@450B@

where R¯1
 MathType@MTEF@5@5@+=feaafiart1ev1aaatCvAUfKttLearuWrP9MDH5MBPbIqV92AaeXatLxBI9gBaebbnrfifHhDYfgasaacPC6xNi=xH8viVGI8Gi=hEeeu0xXdbba9frFj0xb9qqpG0dXdb9aspeI8k8fiI+fsY=rqGqVepae9pg0db9vqaiVgFr0xfr=xfr=xc9adbaqaaeGacaGaaiaabeqaaeqabiWaaaGcbaGafmOuaiLbaebadaWgaaWcbaGaeGymaedabeaaaaa@2E34@, R¯2
 MathType@MTEF@5@5@+=feaafiart1ev1aaatCvAUfKttLearuWrP9MDH5MBPbIqV92AaeXatLxBI9gBaebbnrfifHhDYfgasaacPC6xNi=xH8viVGI8Gi=hEeeu0xXdbba9frFj0xb9qqpG0dXdb9aspeI8k8fiI+fsY=rqGqVepae9pg0db9vqaiVgFr0xfr=xfr=xc9adbaqaaeGacaGaaiaabeqaaeqabiWaaaGcbaGafmOuaiLbaebadaWgaaWcbaGaeGOmaidabeaaaaa@2E36@ are the respective means of the distributions of subjective acceptable resources. After algebraic manipulations we get

V2−V1=∫Rm1Rm2R⋅(R¯1+R¯2−R)⋅D(R)dR
MathType@MTEF@5@5@+=feaafiart1ev1aaatCvAUfKttLearuWrP9MDH5MBPbIqV92AaeXatLxBI9gBaebbnrfifHhDYfgasaacPC6xNi=xI8qiVKYPFjYdHaVhbbf9v8qqaqFr0xc9vqFj0dXdbba91qpepeI8k8fiI+fsY=rqGqVepae9pg0db9vqaiVgFr0xfr=xfr=xc9adbaqaaeGacaGaaiaabeqaaeqabiWaaaGcbaGaemOvay1aaSbaaSqaaiabikdaYaqabaGccqGHsislcqWGwbGvdaWgaaWcbaGaeGymaedabeaakiabg2da9maapehabaGaemOuaiLaeyyXICTaeiikaGIafmOuaiLbaebadaWgaaWcbaGaeGymaedabeaakiabgUcaRiqbdkfaszaaraWaaSbaaSqaaiabikdaYaqabaGccqGHsislcqWGsbGucqGGPaqkcqGHflY1cqWGebarcqGGOaakcqWGsbGucqGGPaqkcqWGKbazcqWGsbGuaSqaaiabdkfasnaaBaaameaacqWGTbqBcqaIXaqmaeqaaaWcbaGaemOuai1aaSbaaWqaaiabd2gaTjabikdaYaqabaaaniabgUIiYdaaaa@5251@

We assume that the minimum acceptable cut-off point *R*_*m *_is such that the acceptable part of the resource distribution includes more than half of the full distribution, i.e., ∫Rm+∞D(R)dR>12
MathType@MTEF@5@5@+=feaafiart1ev1aaatCvAUfKttLearuWrP9MDH5MBPbIqV92AaeXatLxBI9gBaebbnrfifHhDYfgasaacPC6xNi=xH8viVGI8Gi=hEeeu0xXdbba9frFj0xb9qqpG0dXdb9aspeI8k8fiI+fsY=rqGqVepae9pg0db9vqaiVgFr0xfr=xfr=xc9adbaqaaeGacaGaaiaabeqaaeqabiWaaaGcbaWaa8qCaeaacqWGebarcqGGOaakcqWGsbGucqGGPaqkcqWGKbazcqWGsbGucqGH+aGpjuaGdaWcaaqaaiabigdaXaqaaiabikdaYaaaaSqaaiabdkfasnaaBaaameaacqWGTbqBaeqaaaWcbaGaey4kaSIaeyOhIukaniabgUIiYdaaaa@3D4D@. It can then be shown that

2⋅R¯>Rm
 MathType@MTEF@5@5@+=feaafiart1ev1aaatCvAUfKttLearuWrP9MDH5MBPbIqV92AaeXatLxBI9gBaebbnrfifHhDYfgasaacPC6xNi=xI8qiVKYPFjYdHaVhbbf9v8qqaqFr0xc9vqFj0dXdbba91qpepeI8k8fiI+fsY=rqGqVepae9pg0db9vqaiVgFr0xfr=xfr=xc9adbaqaaeGacaGaaiaabeqaaeqabiWaaaGcbaGaeGOmaiJaeyyXICTafmOuaiLbaebacqGH+aGpcqWGsbGudaWgaaWcbaGaemyBa0gabeaaaaa@3466@

where R¯
 MathType@MTEF@5@5@+=feaafiart1ev1aaatCvAUfKttLearuWrP9MDH5MBPbIqV92AaeXatLxBI9gBaebbnrfifHhDYfgasaacPC6xNi=xH8viVGI8Gi=hEeeu0xXdbba9frFj0xb9qqpG0dXdb9aspeI8k8fiI+fsY=rqGqVepae9pg0db9vqaiVgFr0xfr=xfr=xc9adbaqaaeGacaGaaiaabeqaaeqabiWaaaGcbaWaa0aaaeaacqWGsbGuaaaaaa@2D11@ is the mean value of the full (not truncated) distribution of resources. Consequently, if *R*_*m*1 _and *R*_*m*2 _are such that the minimum acceptable cut-off point is less than the mean value of the full resource distribution, and *R*_*m*1 _<*R*_*m*2 _then

∫Rm1+∞D(R)dR>∫Rm2+∞D(R)dR>12
MathType@MTEF@5@5@+=feaafiart1ev1aaatCvAUfKttLearuWrP9MDH5MBPbIqV92AaeXatLxBI9gBaebbnrfifHhDYfgasaacPC6xNi=xI8qiVKYPFjYdHaVhbbf9v8qqaqFr0xc9vqFj0dXdbba91qpepeI8k8fiI+fsY=rqGqVepae9pg0db9vqaiVgFr0xfr=xfr=xc9adbaqaaeGacaGaaiaabeqaaeqabiWaaaGcbaWaa8qCaeaacqWGebarcqGGOaakcqWGsbGucqGGPaqkcqWGKbazcqWGsbGuaSqaaiabdkfasnaaBaaameaacqWGTbqBcqaIXaqmaeqaaaWcbaGaey4kaSIaeyOhIukaniabgUIiYdGccqGH+aGpdaWdXbqaaiabdseaejabcIcaOiabdkfasjabcMcaPiabdsgaKjabdkfasbWcbaGaemOuai1aaSbaaWqaaiabd2gaTjabikdaYaqabaaaleaacqGHRaWkcqGHEisPa0Gaey4kIipakiabg6da+KqbaoaalaaabaGaeGymaedabaGaeGOmaidaaaaa@4E8B@

R¯1>R¯2
 MathType@MTEF@5@5@+=feaafiart1ev1aaatCvAUfKttLearuWrP9MDH5MBPbIqV92AaeXatLxBI9gBaebbnrfifHhDYfgasaacPC6xNi=xI8qiVKYPFjYdHaVhbbf9v8qqaqFr0xc9vqFj0dXdbba91qpepeI8k8fiI+fsY=rqGqVepae9pg0db9vqaiVgFr0xfr=xfr=xc9adbaqaaeGacaGaaiaabeqaaeqabiWaaaGcbaGafmOuaiLbaebadaWgaaWcbaGaeGymaedabeaakiabg6da+iqbdkfaszaaraWaaSbaaSqaaiabikdaYaqabaaaaa@31F7@

and consequently

R¯1+R¯2−R>0
 MathType@MTEF@5@5@+=feaafiart1ev1aaatCvAUfKttLearuWrP9MDH5MBPbIqV92AaeXatLxBI9gBaebbnrfifHhDYfgasaacPC6xNi=xI8qiVKYPFjYdHaVhbbf9v8qqaqFr0xc9vqFj0dXdbba91qpepeI8k8fiI+fsY=rqGqVepae9pg0db9vqaiVgFr0xfr=xfr=xc9adbaqaaeGacaGaaiaabeqaaeqabiWaaaGcbaGafmOuaiLbaebadaWgaaWcbaGaeGymaedabeaakiabgUcaRiqbdkfaszaaraWaaSbaaSqaaiabikdaYaqabaGccqGHsislcqWGsbGucqGH+aGpcqaIWaamaaa@35EB@

if *R*_*m*1 _≤ *R *≤ *R*_*m*2_.

It follows from (3) and (7) that

V_2 _– V_1 _> 0.

**Figure 2:**
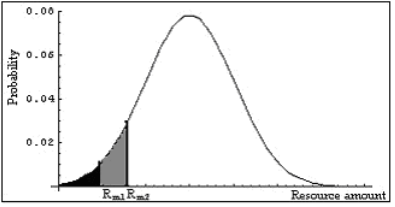
**The distribution of resources in two environments having the same objective resource distribution**. The resource amounts *R*_*m*1 _and *R*_*m*2 _are the minimal acceptable amounts of resources in the two environments. The shaded areas are the parts of the distributions ignored by animals living in the two environments. The larger the ignored area the higher is the subjective variance of the distribution, with the condition that the ignored area is smaller than half of the area below the curve of the distribution.

Thus in a more adverse environment the individual experiences higher subjective variance (and therefore greater uncertainty) in its resources (Figure 2) (see also Appendix 1). The above discussion applies to the case when environmental adversity is measured in terms of an environmental quality (e.g., temperature, predation risk) different from the quality (usually a resource) in terms of which we measure the increased subjective environmental uncertainty. In this case the objective distributions of the second quality, resources, are the same in both environments. If the same environmental quality is used to measure both adversity and uncertainty we arrive at the same conclusion.

We have been dealing so far with subjective uncertainties that are naturally difficult to measure. For several reasons it would be preferable to deal with objective measures of uncertainty. First, it is objective measures that are employed in the following model. Second, as outlined above, we also wish to understand the possible *direct *effects of environmental uncertainty – as well as adversity – in enhancing cooperation. Last, if our conclusions are to be tested, objective measures of uncertainty will probably be required. Now, if the objective uncertainties of two environments differ, while the adversity (i.e., the mean value) of each is the same, then their consequent subjective uncertainties, as defined by a common minimal acceptable cut-off point, will differ in the same direction. This is because the proportion of the distribution that is unacceptable (and thus equated with zero) increases with objective variance. So, objectively more uncertain environments with the same mean expectations are also subjectively more uncertain, and objective uncertainty can stand proxy for subjective uncertainty in the model that follows.

### Uncertainty and cooperation: an agent-based simulation

We have shown how increased adversity leads to increased subjective uncertainty, as measured by subjective variance. We have also shown that subjective and objective variance will be positively correlated. We now go on to show that an increase in objective variance in resources enhances cooperation. It will therefore follow that an increase in the subjective variance of resources will also enhance cooperation. Finally, since adversity causes an increase in subjective uncertainty we can conclude that adversity will favour cooperation.

Using a Prisoner's Dilemma type game theory model we built an agent-based simulation [40] to study the dynamics of the level of cooperation in relation to resource uncertainty. Our agents are generalised organisms that own resources (*R*) that they spend on living costs and use to generate new resources for the future. If the agent has less resource than the amount of living costs the agent dies. The agents live in a continuous two-dimensional world (i.e. unlimited flat continuous space, not a grid), each having a position (*x*, *y*) and change location by random movements, i.e. (*x*_*new*_, *y*_*new*_) = (*x*, *y*) + (*ξ*_*x*_, *ξ*_*y*_), where *ξ*_*x*_, *ξ*_*y *_are small random numbers. The agents have an inclination toward cooperation or competition, expressed as *p *the probability of cooperation of the agent with another agent. If *p *< 0.5 they are more likely to compete than to cooperate. They select their behaviour for each interaction in a probabilistic manner biased by their inclination. This is done by choosing a random number *q *from a uniform distribution over [0,1]; if *q *<*p *they cooperate, otherwise they compete.

Objective resource uncertainty was implemented as the variance of the resource distribution that is used to determine the new amounts of resources of the agents. In each time unit (the time is discrete in our simulation), each agent randomly chooses an interaction partner from its neighbourhood and the partners decide whether to cooperate or compete. The neighbourhood of an agent consists of the ten closest agents, where closeness is measured in terms of spatial distance between agents. The new resource amounts for the agents are determined by taking a sample from a normal resource distribution *N*(X¯
 MathType@MTEF@5@5@+=feaafiart1ev1aaatCvAUfKttLearuWrP9MDH5MBPbIqV92AaeXatLxBI9gBaebbnrfifHhDYfgasaacPC6xNi=xH8viVGI8Gi=hEeeu0xXdbba9frFj0xb9qqpG0dXdb9aspeI8k8fiI+fsY=rqGqVepae9pg0db9vqaiVgFr0xfr=xfr=xc9adbaqaaeGacaGaaiaabeqaaeqabiWaaaGcbaWaa0aaaeaacqWGybawaaaaaa@2D1D@, *σ*_*X*_. The mean value X¯
 MathType@MTEF@5@5@+=feaafiart1ev1aaatCvAUfKttLearuWrP9MDH5MBPbIqV92AaeXatLxBI9gBaebbnrfifHhDYfgasaacPC6xNi=xH8viVGI8Gi=hEeeu0xXdbba9frFj0xb9qqpG0dXdb9aspeI8k8fiI+fsY=rqGqVepae9pg0db9vqaiVgFr0xfr=xfr=xc9adbaqaaeGacaGaaiaabeqaaeqabiWaaaGcbaWaa0aaaeaacqWGybawaaaaaa@2D1D@ is determined by the amount of owned resources according to the payoff table shown in Table 1. The variance *σ*_*X *_is the objective resource uncertainty characterising the environment. Varying the value of *σ*_*X *_allows us to investigate how the level of cooperation responds to the environmental uncertainty.

**Table 1:** The pay-off matrix for the cooperation/competition game

X¯ MathType@MTEF@5@5@+=feaafiart1ev1aaatCvAUfKttLearuWrP9MDH5MBPbIqV92AaeXatLxBI9gBaebbnrfifHhDYfgasaacPC6xNi=xH8viVGI8Gi=hEeeu0xXdbba9frFj0xb9qqpG0dXdb9aspeI8k8fiI+fsY=rqGqVepae9pg0db9vqaiVgFr0xfr=xfr=xc9adbaqaaeGacaGaaiaabeqaaeqabiWaaaGcbaWaa0aaaeaacqWGybawaaaaaa@2D1D@	Cooperate	Compete
Cooperate	f(R1)+Δ2,f(R2)+Δ2 MathType@MTEF@5@5@+=feaafiart1ev1aaatCvAUfKttLearuWrP9MDH5MBPbIqV92AaeXatLxBI9gBaebbnrfifHhDYfgasaacPC6xNi=xH8viVGI8Gi=hEeeu0xXdbba9frFj0xb9qqpG0dXdb9aspeI8k8fiI+fsY=rqGqVepae9pg0db9vqaiVgFr0xfr=xfr=xc9adbaqaaeGacaGaaiaabeqaaeqabiWaaaGcbaGaemOzay2aaeWaaeaacqWGsbGudaWgaaWcbaGaeGymaedabeaaaOGaayjkaiaawMcaaiabgUcaRKqbaoaalaaabaGaeuiLdqeabaGaeGOmaidaaOGaeiilaWIaemOzay2aaeWaaeaacqWGsbGudaWgaaWcbaGaeGOmaidabeaaaOGaayjkaiaawMcaaiabgUcaRKqbaoaalaaabaGaeuiLdqeabaGaeGOmaidaaaaa@3ED1@	*α *· *f*(*R*_1_), *f*(*R*_2_) + Δ
Compete	*f*(*R*_1_) + Δ, *α *· *f*(*R*_2_)	*f*(*R*_1_), *f*(*R*_2_)

Entries indicate the mean of the payoff distribution to the row player followed by the column player. R_1 _and R_2 _are the amount of resources owned by the row and column player respectively, and Δ = [*f*(*R*_1 _+ *R*_2_) - *f*(*R*_1_) - *f*(*R*_2_)]_+ _(i.e., it takes only the positive values of the expression in brackets and it is zero if the value of the expression is negative). The function *f *is a diminishing return function, and R_1 _and R_2 _are typically in the range where 2*f*(*x*) ≤ *f*(2*x*), and 0 <*α *< 1.

The agents produce offspring asexually at the end of their lifetime (unless they die because of lack of resources to cover living costs) who inherit their parent's inclination toward cooperation with some small random change (i.e., *p*_*offspring *_= *p*_*parent *_+ *ξ*, *ξ *∈ [-*ε*, *ε *], and *ε *is a small number, e.g., *ε *= 0.025). The number of offspring depends on the amount of resources of the agent according to the equation

n=α⋅R−(R¯−β⋅σR)σR+n0
 MathType@MTEF@5@5@+=feaafiart1ev1aaatCvAUfKttLearuWrP9MDH5MBPbIqV92AaeXatLxBI9gBaebbnrfifHhDYfgasaacPC6xNi=xI8qiVKYPFjYdHaVhbbf9v8qqaqFr0xc9vqFj0dXdbba91qpepeI8k8fiI+fsY=rqGqVepae9pg0db9vqaiVgFr0xfr=xfr=xc9adbaqaaeGacaGaaiaabeqaaeqabiWaaaGcbaGaemOBa4Maeyypa0dcciGae8xSdeMaeyyXICDcfa4aaSaaaeaacqWGsbGucqGHsislcqGGOaakcuWGsbGugaqeaiabgkHiTiab=j7aIjabgwSixlab=n8aZnaaBaaabaGaemOuaifabeaacqGGPaqkaeaacqWFdpWCdaWgaaqaaiabdkfasbqabaaaaOGaey4kaSIaemOBa42aaSbaaSqaaiabicdaWaqabaaaaa@4681@

where R¯
 MathType@MTEF@5@5@+=feaafiart1ev1aaatCvAUfKttLearuWrP9MDH5MBPbIqV92AaeXatLxBI9gBaebbnrfifHhDYfgasaacPC6xNi=xH8viVGI8Gi=hEeeu0xXdbba9frFj0xb9qqpG0dXdb9aspeI8k8fiI+fsY=rqGqVepae9pg0db9vqaiVgFr0xfr=xfr=xc9adbaqaaeGacaGaaiaabeqaaeqabiWaaaGcbaWaa0aaaeaacqWGsbGuaaaaaa@2D11@ is the mean and *σ*_*R *_is the variance of the owned resources in the population of agents, and *α*, *β*, *n*_0 _are parameters. The offspring share equally the resources of their parent. The offspring start their life from their parent's last location with minor random changes, implying that the offspring of each agent will be closely packed at the beginning. The cluster of offspring diffuses with time, as the offspring make their random movements.

**Figure 3:**
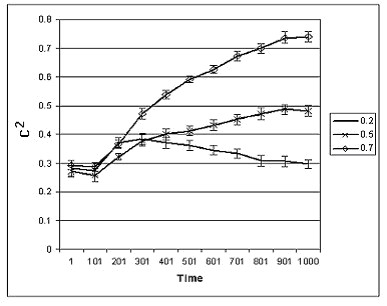
**The evolution of the proportion of cooperating agents**. The graphs show the mean (± SD) values of *c*^2 ^(the proportion of agents participating in cooperation-cooperation interactions, where *c *is the proportion of cooperating agents in the population) in environments with various levels of uncertainty (see the numbers in the box).

We simulated the evolution of 20 agent populations at each of three levels of environmental uncertainty. Each population started with around 1500 individuals (1500 ± a small random element) and the simulation ran for 1000 time units, the agents' mean lifetime being 60 time units. The inclination toward cooperation of the agents was set randomly according to a uniform distribution over [0,1]. We calculated for each simulation, for each time unit, the proportion of agents cooperating in cooperation-cooperation and cooperation-competition interactions. We focused on the proportion of agents participating in cooperation-cooperation interactions, which corresponds to *c*^2^, where *c *is the proportion of cooperating agents in the population. We found that as the objective resource uncertainty level increases, the stable level of cooperation in surviving populations also increases (Figure 3). We note that in the simulations the likelihood of cooperation is increased among clustered kin agents, which is due to the fact that the offspring of an agent start their life as a cluster of agents situated close to their parent's earlier location. However, as the agents follow their diffusion movement (a random walk) the clustering of kin agents is reduced.

The results of our simulation study show that a high level of objective resource uncertainty induces a high level of cooperation in agent populations. We have no analytical results that would explain our simulation results unambiguously. One possibility is that the payoff matrices of games played deviate from the Prisoner's Dilemma matrix, and this causes more cooperation as environmental risk increases [41]. Analysing our payoff matrix (see Table 1) we can exclude this possibility. Our payoff matrix is constructed such that in all cases the Prisoner's Dilemma inequalities are satisfied, i.e., payoff(cheater) > payoff (cooperation) > payoff(joint non-cooperation) > payoff (sucker), and payoff(cooperation) > [(payoff(cheater) + payoff(sucker)]/2, if Δ>0, and we have equalities instead of inequalities if Δ = 0, except the case of payoff(joint non-cooperation) > payoff (sucker), which is always satisfied. The payoff generating function *f *is set such that in most cases Δ>0, although the proportion of cases with Δ = 0 increases as environmental risk increases.

In our view the phenomenological explanation is that populations of agents survive in an uncertain environment only if the experienced individual uncertainties are around a steady-state level. At the steady-state level of experienced uncertainty the population maintains its size without major variations. By cooperation agents share individually perceived uncertainties, reducing their actual experienced uncertainty (similar to the reduction of risk on joining an insurance scheme). Higher environmental uncertainty implies that more cooperation is needed to reduce experienced individual uncertainties of agents in order to keep these uncertainties around the steady-state level. According to this argument, higher environmental uncertainty implies the presence of more cooperation in surviving populations, which is in good agreement with our simulation findings. Some research works suggest that above random level segregation of cooperating and non-cooperating agents is a key mechanism in the development of populations with high level of cooperation [14,42-44]. In our case, as we noted above, the offspring of reproducing agents form originally a cluster around the earlier location of their parent, and later this cluster diffuses as the agent follow their random walk. This indicates that possibly the segregation mechanism contributes to some extent in our simulation to the emergence of agent populations that have sufficient level of cooperation that makes the experienced uncertainty of agents stay around the steady-state level.

## Conclusion

The simulation study captures the essential features of the natural examples: the positive impact of resource adversity or uncertainty on cooperation. As our simulation is based on a very limited set of assumptions, the results are likely to be of broad applicability and may explain why cooperation flourishes in risky environments, whether risk is conceptualised as adversity, objective environmental variance or subjective environmental uncertainty [17]. Elsewhere we demonstrate another way in which adversity and uncertainty may enhance cooperation [32].

Social psychological influences of perceived threat on group solidarity in humans [26-28] may seem removed from conclusions concerning cooperative behaviour, and outside the explanatory scope of models based on fitness considerations. However, human cooperative tendencies, and the associated prosocial cognitions and affective states, evolved in the small groups of early human societies [32,45] – or earlier – where social responses to environmental adversity [27] and uncertainty [31] will have impacted directly on fitness. We should therefore expect to find our predicted relationships between perceived risk and behaviours broadly classifiable as cooperative in contemporary societies, as long as these contemporary perceptions and behaviours are recognized and mediated by the putative evolved cognitive mechanisms.

## Methods

The methods are described in the main body of this paper (Results and discussion section).

## Appendix

We show, using an alternative route, that the subjective variance of resources increases monotonically with the increase of environmental adversity if the acceptable part of the resource distribution includes more than half of the full distribution, i.e.,

∫Rm+∞D(R)dR>12
MathType@MTEF@5@5@+=feaafiart1ev1aaatCvAUfKttLearuWrP9MDH5MBPbIqV92AaeXatLxBI9gBaebbnrfifHhDYfgasaacPC6xNi=xI8qiVKYPFjYdHaVhbbf9v8qqaqFr0xc9vqFj0dXdbba91qpepeI8k8fiI+fsY=rqGqVepae9pg0db9vqaiVgFr0xfr=xfr=xc9adbaqaaeGacaGaaiaabeqaaeqabiWaaaGcbaWaa8qCaeaacqWGebarcqGGOaakcqWGsbGucqGGPaqkcqWGKbazcqWGsbGucqGH+aGpjuaGdaWcaaqaaiabigdaXaqaaiabikdaYaaaaSqaaiabdkfasnaaBaaameaacqWGTbqBaeqaaaWcbaGaey4kaSIaeyOhIukaniabgUIiYdaaaa@3D9B@

where *R*_*m *_is the minimum acceptable cut-off point.

Define Rm¯
 MathType@MTEF@5@5@+=feaafiart1ev1aaatCvAUfKttLearuWrP9MDH5MBPbIqV92AaeXatLxBI9gBaebbnrfifHhDYfgasaacPC6xNi=xH8viVGI8Gi=hEeeu0xXdbba9frFj0xb9qqpG0dXdb9aspeI8k8fiI+fsY=rqGqVepae9pg0db9vqaiVgFr0xfr=xfr=xc9adbaqaaeGacaGaaiaabeqaaeqabiWaaaGcbaWaa0aaaeaacqWGsbGudaWgaaWcbaGaemyBa0gabeaaaaaaaa@2EA0@ as the subjective mean of the resource distribution:

Rm¯=∫Rm+∞RD(R)dR=∫Rm+∞RD(R)dR+∫0RmR⋅δ0dR
MathType@MTEF@5@5@+=feaafiart1ev1aaatCvAUfKttLearuWrP9MDH5MBPbIqV92AaeXatLxBI9gBaebbnrfifHhDYfgasaacPC6xNi=xI8qiVKYPFjYdHaVhbbf9v8qqaqFr0xc9vqFj0dXdbba91qpepeI8k8fiI+fsY=rqGqVepae9pg0db9vqaiVgFr0xfr=xfr=xc9adbaqaaeGacaGaaiaabeqaaeqabiWaaaGcbaWaa0aaaeaacqWGsbGudaWgaaWcbaGaemyBa0gabeaaaaGccqGH9aqpdaWdXbqaaiabdkfasjabdseaejabcIcaOiabdkfasjabcMcaPiabdsgaKjabdkfasbWcbaGaemOuai1aaSbaaWqaaiabd2gaTbqabaaaleaacqGHRaWkcqGHEisPa0Gaey4kIipakiabg2da9maapehabaGaemOuaiLaemiraqKaeiikaGIaemOuaiLaeiykaKIaemizaqMaemOuaifaleaacqWGsbGudaWgaaadbaGaemyBa0gabeaaaSqaaiabgUcaRiabg6HiLcqdcqGHRiI8aOGaey4kaSYaa8qCaeaacqWGsbGucqGHflY1iiGacqWF0oazdaWgaaWcbaGaeGimaadabeaakiabdsgaKjabdkfasbWcbaGaeGimaadabaGaemOuai1aaSbaaWqaaiabd2gaTbqabaaaniabgUIiYdaaaa@5F11@

where *δ*_0 _is a Dirac-delta function centred on 0 such that ∫0Rmδ0dR=∫0RmD(R)dR
MathType@MTEF@5@5@+=feaafiart1ev1aaatCvAUfKttLearuWrP9MDH5MBPbIqV92AaeXatLxBI9gBaebbnrfifHhDYfgasaacPC6xNi=xH8viVGI8Gi=hEeeu0xXdbba9frFj0xb9qqpG0dXdb9aspeI8k8fiI+fsY=rqGqVepae9pg0db9vqaiVgFr0xfr=xfr=xc9adbaqaaeGacaGaaiaabeqaaeqabiWaaaGcbaWaa8qCaeaaiiGacqWF0oazdaWgaaWcbaGaeGimaadabeaakiabdsgaKjabdkfasbWcbaGaeGimaadabaGaemOuai1aaSbaaWqaaiabd2gaTbqabaaaniabgUIiYdGccqGH9aqpdaWdXbqaaiabdseaejabcIcaOiabdkfasjabcMcaPiabdsgaKjabdkfasbWcbaGaeGimaadabaGaemOuai1aaSbaaWqaaiabd2gaTbqabaaaniabgUIiYdaaaa@44C7@.

Writing the subjective variance as a function of *R*_*m *_we get

V(Rm)=∫Rm+∞R2D(R)dR−Rm¯2
MathType@MTEF@5@5@+=feaafiart1ev1aaatCvAUfKttLearuWrP9MDH5MBPbIqV92AaeXatLxBI9gBaebbnrfifHhDYfgasaacPC6xNi=xI8qiVKYPFjYdHaVhbbf9v8qqaqFr0xc9vqFj0dXdbba91qpepeI8k8fiI+fsY=rqGqVepae9pg0db9vqaiVgFr0xfr=xfr=xc9adbaqaaeGacaGaaiaabeqaaeqabiWaaaGcbaGaemOvayLaeiikaGIaemOuai1aaSbaaSqaaiabd2gaTbqabaGccqGGPaqkcqGH9aqpdaWdXbqaaiabdkfasnaaCaaaleqabaGaeGOmaidaaOGaemiraqKaeiikaGIaemOuaiLaeiykaKIaemizaqMaemOuaifaleaacqWGsbGudaWgaaadbaGaemyBa0gabeaaaSqaaiabgUcaRiabg6HiLcqdcqGHRiI8aOGaeyOeI0Yaa0aaaeaacqWGsbGudaWgaaWcbaGaemyBa0gabeaaaaGcdaahaaWcbeqaaiabikdaYaaaaaa@4809@

We calculate the derivative of the subjective variance with respect to the minimum cut-off point *R*_*m*_

∂∂RmV(Rm)=∂∂Rm∫Rm+∞R2D(R)dR−∂∂Rm(∫Rm+∞RD(R)dR)2=2RmD(Rm)Rm¯−Rm2D(Rm)=RmD(Rm)⋅(2Rm¯−Rm)
MathType@MTEF@5@5@+=feaafiart1ev1aaatCvAUfKttLearuWrP9MDH5MBPbIqV92AaeXatLxBI9gBaebbnrfifHhDYfgasaacPC6xNi=xI8qiVKYPFjYdHaVhbbf9v8qqaqFr0xc9vqFj0dXdbba91qpepeI8k8fiI+fsY=rqGqVepae9pg0db9vqaiVgFr0xfr=xfr=xc9adbaqaaeGacaGaaiaabeqaaeqabiWaaaGceaqabeaajuaGdaWcaaqaaiabgkGi2cqaaiabgkGi2kabdkfasnaaBaaabaGaemyBa0gabeaaaaGccqWGwbGvcqGGOaakcqWGsbGudaWgaaWcbaGaemyBa0gabeaakiabcMcaPiabg2da9KqbaoaalaaabaGaeyOaIylabaGaeyOaIyRaemOuai1aaSbaaeaacqWGTbqBaeqaaaaakmaapehabaGaemOuai1aaWbaaSqabeaacqaIYaGmaaGccqWGebarcqGGOaakcqWGsbGucqGGPaqkcqWGKbazcqWGsbGuaSqaaiabdkfasnaaBaaameaacqWGTbqBaeqaaaWcbaGaey4kaSIaeyOhIukaniabgUIiYdGccqGHsisldaWcaaqaaiabgkGi2cqaaiabgkGi2kabdkfasnaaBaaaleaacqWGTbqBaeqaaaaakmaabmaabaWaa8qCaeaacqWGsbGucqWGebarcqGGOaakcqWGsbGucqGGPaqkcqWGKbazcqWGsbGuaSqaaiabdkfasnaaBaaameaacqWGTbqBaeqaaaWcbaGaey4kaSIaeyOhIukaniabgUIiYdaakiaawIcacaGLPaaadaahaaWcbeqaaiabikdaYaaakiabg2da9aqaaiabikdaYiabdkfasnaaBaaaleaacqWGTbqBaeqaaOGaemiraqKaeiikaGIaemOuai1aaSbaaSqaaiabd2gaTbqabaGccqGGPaqkdaqdaaqaaiabdkfasnaaBaaaleaacqWGTbqBaeqaaaaakiabgkHiTiabdkfasnaaDaaaleaacqWGTbqBaeaacqaIYaGmaaGccqWGebarcqGGOaakcqWGsbGudaWgaaWcbaGaemyBa0gabeaakiabcMcaPiabg2da9iabdkfasnaaBaaaleaacqWGTbqBaeqaaOGaemiraqKaeiikaGIaemOuai1aaSbaaSqaaiabd2gaTbqabaGccqGGPaqkcqGHflY1cqGGOaakcqaIYaGmdaqdaaqaaiabdkfasnaaBaaaleaacqWGTbqBaeqaaaaakiabgkHiTiabdkfasnaaBaaaleaacqWGTbqBaeqaaOGaeiykaKcaaaa@93FB@

If condition (A1) is satisfied it can be shown that

2Rm¯>Rm
 MathType@MTEF@5@5@+=feaafiart1ev1aaatCvAUfKttLearuWrP9MDH5MBPbIqV92AaeXatLxBI9gBaebbnrfifHhDYfgasaacPC6xNi=xI8qiVKYPFjYdHaVhbbf9v8qqaqFr0xc9vqFj0dXdbba91qpepeI8k8fiI+fsY=rqGqVepae9pg0db9vqaiVgFr0xfr=xfr=xc9adbaqaaeGacaGaaiaabeqaaeqabiWaaaGcbaGaeGOmaiZaa0aaaeaacqWGsbGudaWgaaWcbaGaemyBa0gabeaaaaGccqGH+aGpcqWGsbGudaWgaaWcbaGaemyBa0gabeaaaaa@33AE@

So, we can conclude that if condition (A1) is satisfied then

∂∂RmV(Rm)>0
 MathType@MTEF@5@5@+=feaafiart1ev1aaatCvAUfKttLearuWrP9MDH5MBPbIqV92AaeXatLxBI9gBaebbnrfifHhDYfgasaacPC6xNi=xI8qiVKYPFjYdHaVhbbf9v8qqaqFr0xc9vqFj0dXdbba91qpepeI8k8fiI+fsY=rqGqVepae9pg0db9vqaiVgFr0xfr=xfr=xc9adbaqaaeGacaGaaiaabeqaaeqabiWaaaGcbaqcfa4aaSaaaeaacqGHciITaeaacqGHciITcqWGsbGudaWgaaqaaiabd2gaTbqabaaaaOGaemOvayLaeiikaGIaemOuai1aaSbaaSqaaiabd2gaTbqabaGccqGGPaqkcqGH+aGpcqaIWaamaaa@39E9@

This means that, given the condition (A1) being satisfied, the variance is an increasing function of the minimum cut-off point value, which increases with the adversity of the environment. This implies that the subjective resource variance of a more adverse environment is larger than the subjective variance of a less adverse environment, if the acceptable part of the resource distribution includes more than half of the full resource distribution.

## Authors' contributions

PA derived the analytical results, developed the simulation environment and ran the simulations, and drafted the versions of the paper. JL provided the review and organization of the background and contributed with critical review and discussion of draft versions. GR contributed with critical review and discussion of draft versions. All authors read and approved the final manuscript.
